# Relationship between Body Mass Composition, Bone Mineral Density, Skin Fibrosis and 25(OH) Vitamin D Serum Levels in Systemic Sclerosis

**DOI:** 10.1371/journal.pone.0137912

**Published:** 2015-09-16

**Authors:** Addolorata Corrado, Ripalta Colia, Angiola Mele, Valeria Di Bello, Antonello Trotta, Anna Neve, Francesco Paolo Cantatore

**Affiliations:** Rheumatology Clinic, Department of Medical and Surgical Sciences, University of Foggia, Foggia, Italy; University of Texas Health Science Center at Houston, UNITED STATES

## Abstract

A reduced bone mineral density (BMD) is observed in several rheumatic autoimmune diseases, including Systemic Sclerosis (SSc); nevertheless, data concerning the possible determinants of bone loss in this disease are not fully investigated. The aim of this study is to evaluate the relationship between BMD, body mass composition, skin sclerosis and serum Vitamin D levels in two subsets of SSc patients. 64 post-menopausal SSc patients, classified as limited cutaneous (lcSSc) or diffuse cutaneous (dcSSc) SSc, were studied. As control, 35 healthy post-menopausal women were recruited. Clinical parameters were evaluated, including the extent of skin involvement. BMD at lumbar spine, hip, femoral neck and body mass composition were determined by dual-energy X-ray absorptiometry. Serum calcium, phosphorus, alkaline phosphatase, urine pyridinium cross-links, intact parathyroid hormone and 25-hydroxyvitamin D (25OHD) were measured. BMD at spine, femoral neck and total hip was significantly lower in SSc patients compared to controls. In dcSSc subset, BMD at spine, femoral neck and total hip was significantly lower compared to lcSSc. No differences in both fat and lean mass were found in the three study groups even if patients with dcSSc showed a slightly lower total body mass compared to healthy controls. Total mineral content was significantly reduced in dSSc compared to both healthy subjects and lcSSc group. Hypovitaminosis D was observed both in healthy post-menopausal women and in SSc patients, but 25OHD levels were significantly lower in dcSSc compared to lcSSc and inversely correlated with the extent of skin thickness. These results support the hypothesis that the extent of skin involvement in SSc patients could be an important factor in determining low circulating levels of 25OHD, which in turn could play a significant role in the reduction of BMD and total mineral content.

## Introduction

Systemic sclerosis (SSc) is a rare chronic autoimmune disease characterised by an increased synthesis and deposition of extra-cellular matrix in skin and internal organs and widespread macro and micro- vascular damage. Depending on the extent of skin fibrosis, SSc can be classified in two main subtypes, the limited cutaneous form (lcSSc) and the diffuse cutaneous form (dcSSc) [1].

Several previous studies showed a reduced bone mineral density (BMD) in patients with SSc [2,3] but data are discordant as the determinants of osteopenia/ospertoporosis in this disease are not well clarified and the knowledge about the exact physio-pathologic mechanism of BMD loss is limited. The factors that may affect BMD in SSc patients are various and include reduced physical activity, corticosteroid and immunosuppressive treatment, malabsorption, systemic inflammation and circulating Vitamin D levels. Vitamin D levels are directly related to BMD in different races and in both gender [4] and hypovitaminosis D is a well recognized causal factor for reduced bone mass, due to its role in regulating bone remodelling, calcium and phosphorous metabolism and mineralization processes.

In the latest years, there has been growing interest concerning the role of Vitamin D both in bone metabolism and in several clinical manifestations of chronic autoimmune diseases [5–7]. Low Vitamin D serum concentrations have been reported in several autoimmune conditions, including rheumatoid arthritis, systemic lupus erythematosus and undifferentiated connective tissue diseases [8–11]. Few studies reported low Vitamin D levels in patients with SSc, but the possible causes of hypovitaminosis D are not fully investigated. There are several possible factors inducing hypovitaminosis D in SSc, including reduced sun exposure and skin fibrosis. Although it has been reported that bone mass in SSc patients was related to the extent of skin involvement [12], no data are available regarding the possible relationship between the degree of skin fibrosis and Vitamin D levels. Furthermore, no correlation between low Vitamin D levels, BMD and body mass composition has been clearly found.

The aim of this study is to evaluate BMD, body mass composition, and Vitamin D levels in two skin subsets (limited or diffuse) of SSc patients and to assess the possible correlation with the extent of skin sclerosis.

## Patients and Methods

64 consecutive post-menopausal SSc patients (mean age 64,5, range 42–75 years) classified according to Leroy as having limited cutaneous systemic sclerosis (lcSSc) or diffuse cutaneous systemic sclerosis (dcSSc), were enrolled in the study. As control group 35 healthy post-menopausal women matched for age (mean age 66,1 range 46–78) and years from menopause were recruited.

For both subsets of patients and healthy controls body mass index (BMI) and menopausal status (age at onset and duration) were assessed. BMI was calculated as body weight (Kg) divided by square of height (m^2^) and thus expressed as Kg/m^2^.

Dual-energy x-ray absorptiometry (DEXA) measurements were performed in all participants using a total body scanner (QDR 4500 Acclaim Series Elite, Hologic Inc., Bedford, MA, USA) to evaluate BMD, body mass composition and T-score. BMD was evaluated both at lumbar spine (L1-L4) and hip (femoral neck and total hip) and was expressed as g/cm^2^; osteoporosis was defined as −2.5 or lower T-score values and osteopenia as -1 to -2.5 T-score values.

Weekly physical activity was assessed by the Rapid Assessment of Physical Activities (RAPA) questionnaire [13].Total, truncal and extremity fat mass (Kg), corresponding lean mass (Kg) and total body bone mineral content (BMC) (Kg) were assessed. Extremity fat mass and lean mass were comprised of both upper and lower limbs. Truncal fat mass and lean mass were comprised of thoracic, abdominal and pelvic regions.

Serum levels of 25 OH Vitamin D (25OHD) were measured using the commercial kit (LIAISON 25-OH Vitamin D assay—DiaSorin), considering as normal a range between 30–100 ng/ml. In all recruited subjects, 25OHD serum concentration was evaluated in the period between June-September. None of the recruited subjects received vitamin D supplementation and other medication, including bisphosphonates, with a specific effect on bone metabolism in the previous year. Laboratory parameters of bone metabolism were measured in all recruited subjects and included serum calcium, phosphorus, alkaline phosphatase, urine pyridinium cross-links, serum intact parathyroid hormone (PTH). All blood samples were analysed at a single laboratory.

For each patient, the extent of skin fibrosis was evaluated by the modified Rodnan Skin Score (RSS), and all patients were evaluated also for internal organ involvement.

Particularly, pulmonary involvement was evaluated by chest radiograph, high resolution CT (HRCT) and pulmonary function test with diffusing lung capacity for carbon monoxide adjusted to haemoglobin (DLCO). Cardiac involvement was assessed by electrocardiogram; all patients were screened for pulmonary arterial hypertension (PAH) with doppler echocardiogram and when appropriate, according to DETECT algorithm [14], selected patients underwent right hearth cardiac catheterism; the diagnosis of PAH was confirmed if mean pulmonary arterial pressure was ≥25 mmHg with pulmonary capillary wedge pressure of ≤15 mmHg. Patients were assessed for gastrointestinal involvement by esophagus radiographic abnormalities and through clinical evaluation (chronic diarrhoea and episodes of functional small intestine obstruction). As in SSc patients malabsorption is mainly caused by small intestinal bacterial overgrowth (SIBO), to evaluate the prevalence of malabsorption we performed the C14 xylose breath test in all recruited SSc patients; further, for each recruited patient, serum levels of vitamin K or, as an alternative prothrombin time, were measured. A diagnosis of SIBO was based on positive C14 xylose breath test, and blood assays were used to evaluate the presence of malabsorption. A condition of malabsorption was defined when the C14 xylose breath test was positive together with the presence of low levels of vitamin K or low values of prothrombin time.

Patients were investigated for renal failure or previous history of scleroderma renal crisis. Musculo-skeletal involvement (arthritis and/or myositis), nailfold video-capillaroscopy, erythrocyte sedimentation rate (ESR), anti-nuclear antibodies (ANA) were also assessed. Corticosteroids treatment (doses and duration) was evaluated.

All the investigations were performed at Hospital as part of routine clinical evaluation of SSc patients. Written informed consent was obtained from all the participants to the study. The study was approved by the Ospedali Riuniti University Hospital’s Ethics Committee for Medical Research (Foggia- Italy) and carried out in accordance with the Helsinki Declaration.

## Statistical Analysis

Continuous variables were reported as means and standard deviations while categorical variables were summarized as percentages.

Differences between groups were assessed using the Student’s t-test for continuous variables. Comparison of percentages between groups was performed by Fisher’s exact test. Pearson correlation coefficient was used to evaluate the correlation between selected continuous variables (25OHD levels, PTH levels and Rodnan skin score, BMD, duration of disease). Multiple logistic regression was used to assess the relationship between selected demographic and clinical variables (internal organ involvement) and BMD values / Vitamin D levels.

Values of p<0.05 were considered to be significant.

## Results

### Demographic findings and menopause status

The demographic and clinic characteristics of the two SSc subgroups (limited and diffuse cutaneous form) are shown in Table 1. Of enrolled SSc patients, 33 were classified as having the limited cutanueous subset of SSc (lcSSc) and 31 were classified as having the diffuse cutaneous subsets of the disease (dcSSc). No significant differences were observed between the two SSc cutaneous variants and control subjects regarding age, the age at menopause, duration of menopause and weekly physical activity.

**Table 1:** Demographic and clinical characteristics of SSc patients and healthy controls.

	Healthy controls	limited SSc	diffuse SSc	p
**Total number**	35	33	31	
**Age (mean ± SD)**	66,1±7,9	63,9±12,5	65,3±8,1	n.s.
**BMI (mean ±SD)**	26,88±4,5	27,61±6,47	25,85±5,73	n.s.
**Anticentromere antibodies (%, n)**		81,8%(27)	12,9%(4)	
**Anti Topoisomerase antibodies (%, n)**		18,2%(6)	87,1%(27)	
**Duration of disease (years)** *		7,3±2,8	8,9±4,2	n.s.
**Modified Rodnan Skin Score**		11,75±7,01	23,4±8,2	p<0,001
**Raynaud phenomenon**		100%(33)	100%(31)	n.s.
**Years from onset of Raynaud phenomenon**		19,3±8,2	21,4±9,8	n.s
**Interstitial lung disease (%,n)**		63,7% (21)	77,4% (24)	p<0,01
**Pulmonary arterial hypertension (%, n)**		48,4% (16)	45,1% (14)	n.s.
**Esophageal dysmotility (%, n)**		87,8% (29)	90,3% (28)	n.s.
**Malabsorption (%, n)**		27,3% (9)	24,2% (8)	n.s.
**Scleroderma renal crisis (%, n)**		0% (0)	0% (0)	n.s.
**Musculo-skeletal involvement (%, n)**		54,5% (18)	61,2% (19)	n.s.
**Years from menopause (mean, SD)**	14,8±7,5	13,9±8,3	15,1±9,9	n.s.

* Years from the onset of the first sign/symptom of diseased other than Raynaud phenomenon

### Bone Mineral Density, Body Mass Index and Body Mass Composition

We found that compared to healthy controls, BMD at spine and total hip was significantly lower in dcSSc patients (0,963 g/cm^2^ in control group vs 0,853 g/cm^2^ in dcSSc at spine, p<0,001; 0,932 g/cm^2^ in control group vs 0,755 g/cm^2^ in dSSc at total hip,p<0,001) whereas there was no significant difference between healthy subjects and lSSc patients. BMD at femoral neck was lower both in dcSSc and lcSSc patients compared to healthy subjects (0,826 g/cm^2^ in control group vs 0,762 g/cm^2^ in lcSSs and 0,656 g/cm^2^ in dcSSs, p <0,05 and p<0,01 respectively)

**Fig 1:**
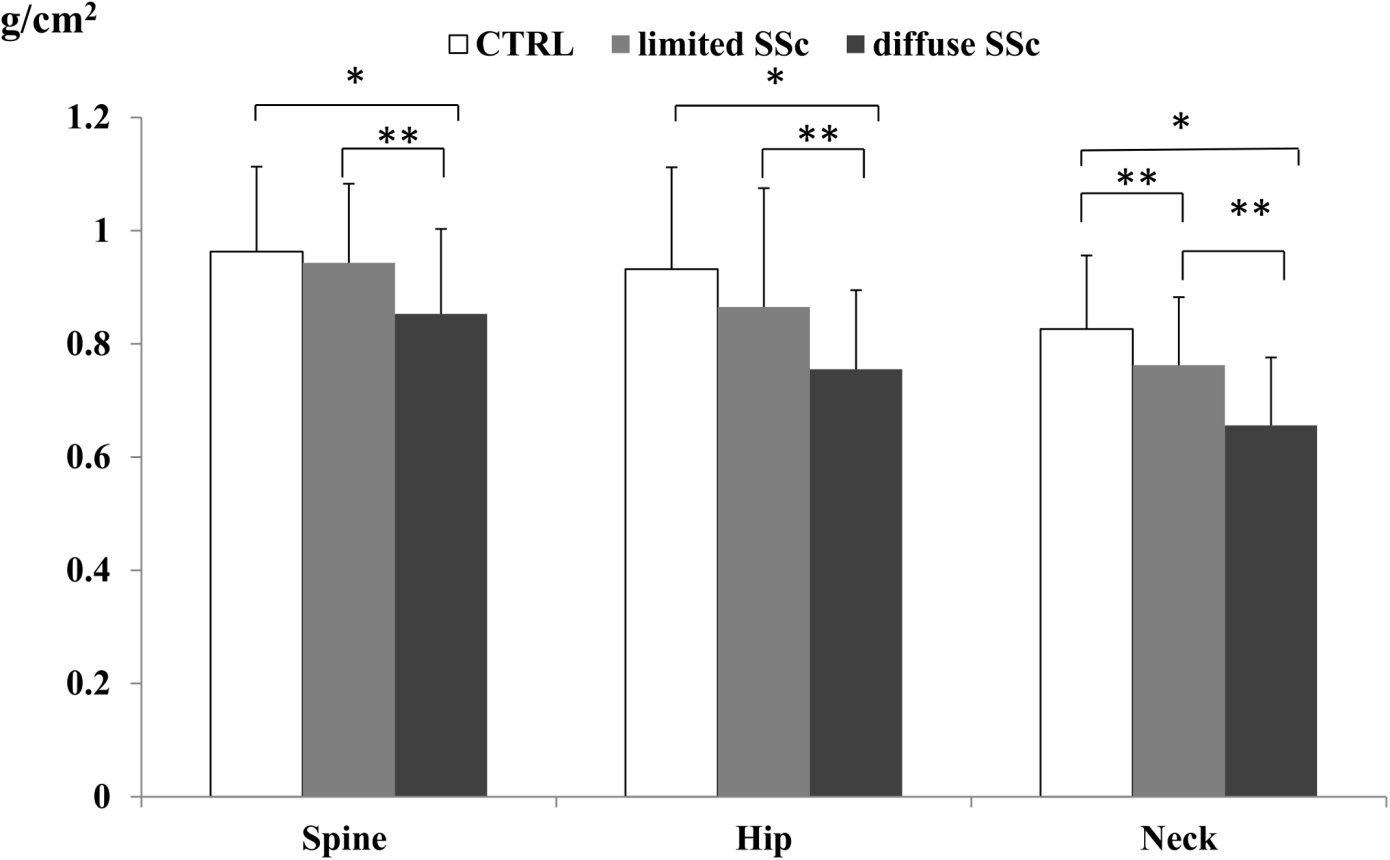
BMD values at spine, femoral neck and total hip in healthy post-menopausal women and patients with limited and diffuse SSc. BMD at spine and total hip is significantly lower in dcSSc patients compared to both healthy subjects and lSSc patients. No significant difference between healthy subjects and lcSSc patients is observed. At femoral neck BMD was significantly lower both in dcSSc and lcSSc patients compared to healthy subjects. Both at spine, total hip and femoral neck, BMD was significantly lower in dcSSc patients compared to lcSSc patients *p<0,001; **p<0,01.

Both at spine, total hip and femoral neck, BMD was significantly lower in dcSSc patients compared to lcSSc patients (Fig 1). T-scores values and percentage of osteoporosis and osteopenia in healthy control and in the two subsets of SSc patients are showed in Table 2.

No differences in BMI were observed between healthy subjects (26,88 Kg/m^2^) and the whole group of SSc patients (26,77 Kg/m^2^) and between the diffuse and limited SSc groups (26, 88 vs 27,61 and 25, 85 Kg/m^2^ respectively).

**Table 2:** T-scores values and prevalence of osteoporosis and osteopenia in healthy controls and SSc patients.

T-scores	Controls	lcSSc	dcSSC
spine	-1,08	-1,26	-2,07
total hip	-0,35	-0,68	-1,83
femoral neck	-0,67	-1,31	-2,37
**Osteoporosis (n, %)**			
spine	3(8,5%)	7(21,2%)	14(45,2%)
total hip	2(5,7%)	3(9,8%)	8(26%)
femoral neck	4(11,4%)	7(21,2%)	16(51,6%)
**Osteopenia (n, %)**			
spine	17(48,6%)	9(27,3%)	7(22,6%)
total hip	12(34,3%)	8(24,2%)	13(42%)
femoral neck	7(20%)	11(33,3%)	11(35,4%)

**Fig 2:**
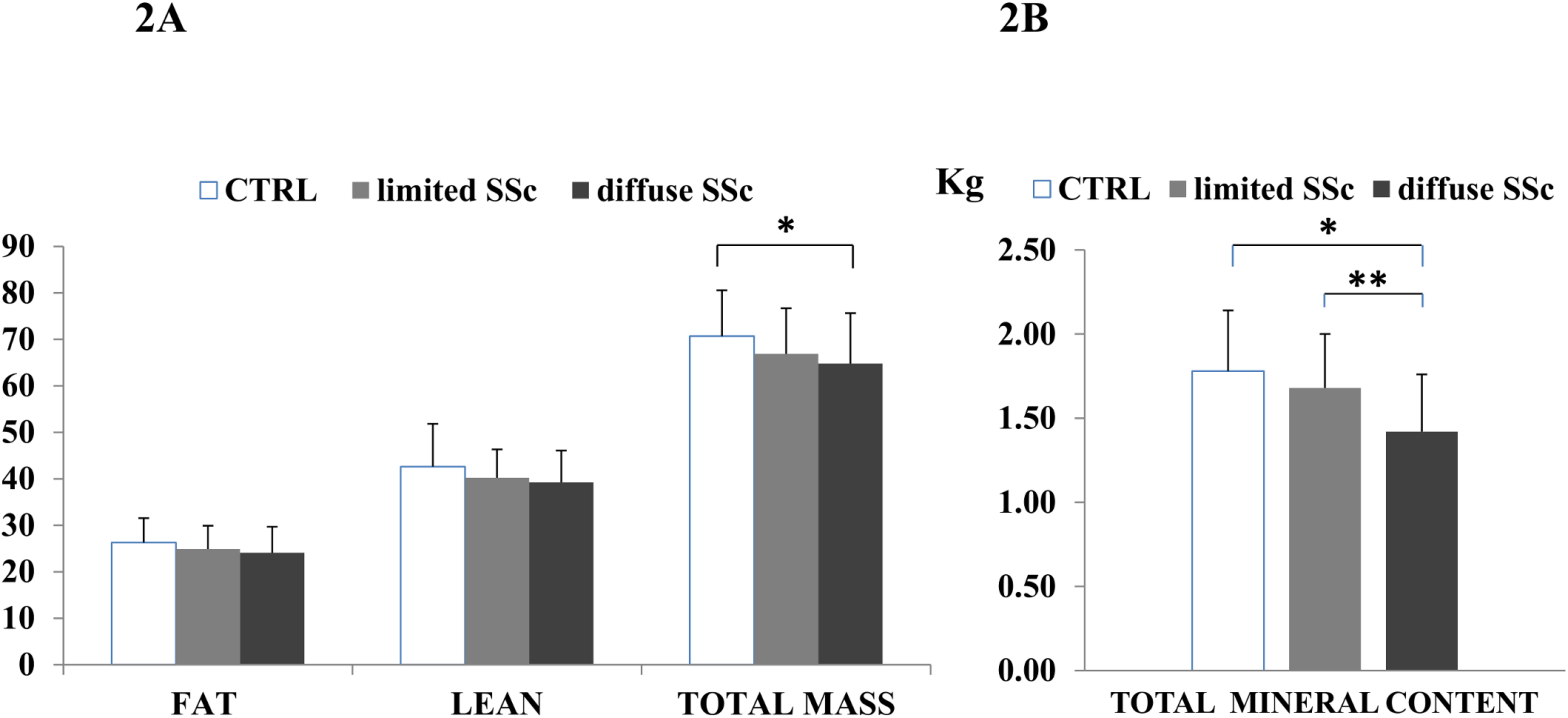
Analysis of body mass composition in healthy post-menopausal women and patients with diffuse and limited SSc subsets (fat, lean and total mass and total mineral content). **2A.** No differences in fat mass and lean mass are observed between healthy control group, limited and diffuse cutaneous subsets of SSc; total body mass was significantly lower in dcSSc patients compared to healthy controls (*p<0,05). Patients with dcSSc also show a slightly lower total body mass compared to lcSSc which do not reach statistical significance. **2B.** No difference in total mineral content is observed between healthy subjects and lcSSc patients, whereas total mineral content was significantly lower in dcSSc compared to both control group (*p<0,001) and lcSSc group (**p<0,01).

Concerning body mass composition, no differences in fat and lean mass were observed between healthy subjects and SSc patients (both lcSSc and dcSSc), whereas total body mass was significantly lower in dSSc patients compared to healthy controls. Patients with dcSSc also showed a slightly lower total body mass compared to lcSSc which did not reach statistical significance (Fig 2A). No difference in total mineral content was observed between healthy subjects and lcSSc patients, whereas total mineral content was significantly lower in dcSSc compared to both control healthy group (p<0,001) and lcSSc group (p<0,01)

### Serum 25 OH Vitamin D and intact PTH levels in limited cutaneous SSc and diffuse cutaneous SSc

**Fig 3:**
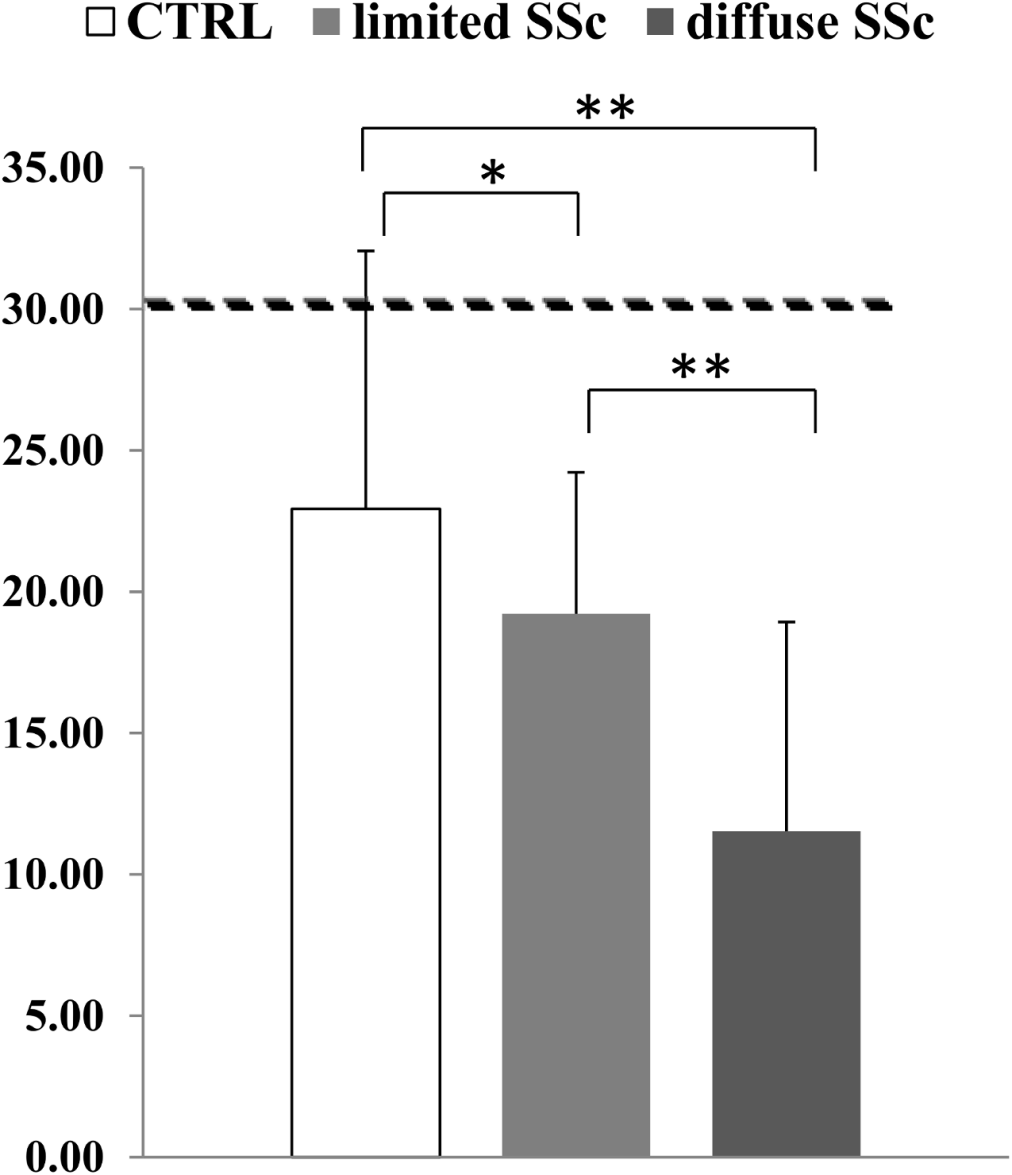
25OHD serum levels are below the lower limit of normal range levels (30 ng/ml, dotted line) both in healthy subjects and the two cutaneous subsets of SSc. Nevertheless in healthy subjects the observed 25OHD circulating levels are significantly higher compared to both lcSSc and dcSSc; furthermore, in patients with dcSSc, 25OHD levels are significantly lower compared to the limited cutaneous form. *p<0,01; **p<0,001.

**Fig 4:**
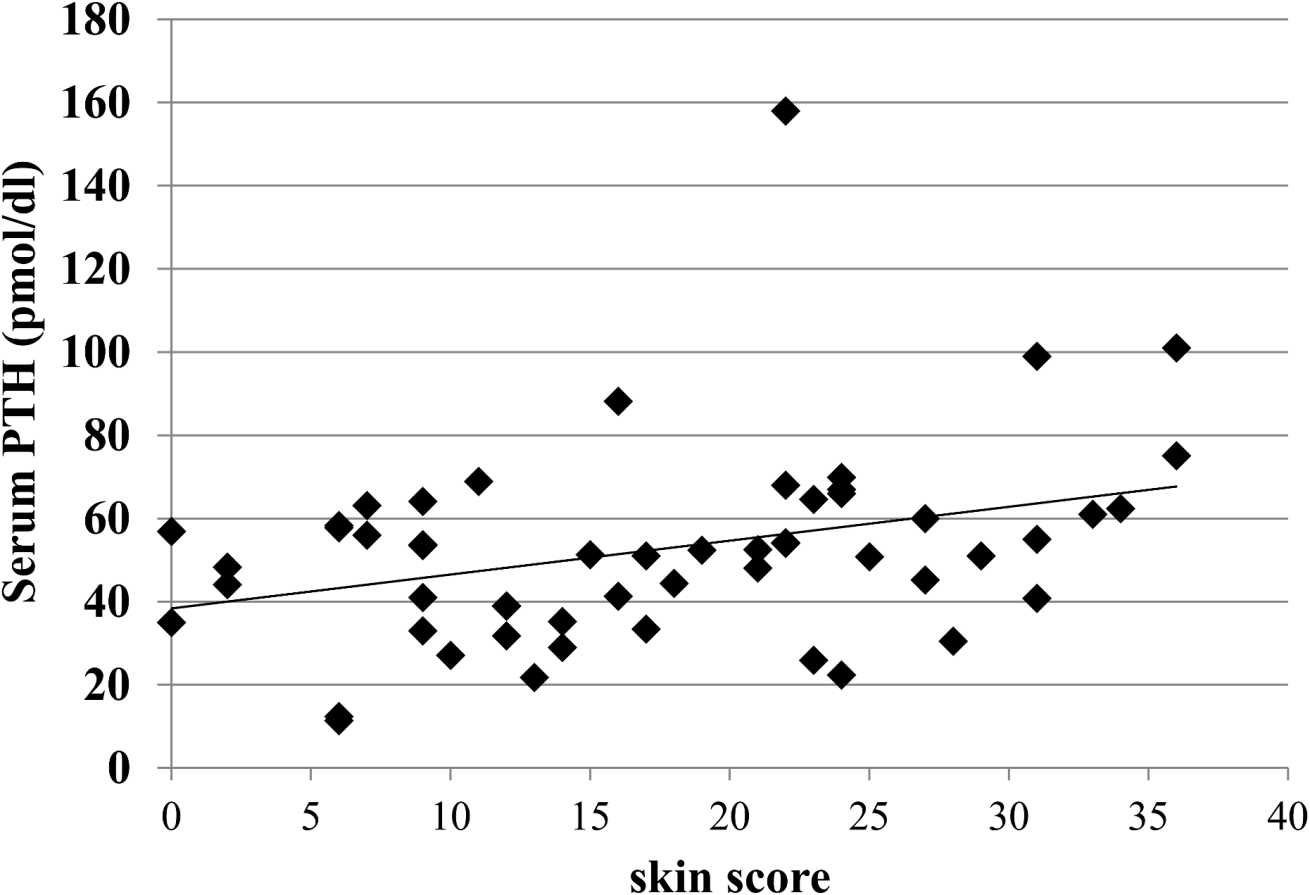
Relationship between the degree of skin fibrosis assessed by modified Rodnan skin score and 25OHD levels in SSc patients. A significant inverse relationship between the degree of skin fibrosis and circulating levels of 25OHD (r = -0,7, p<0,05).

**Fig 5:**
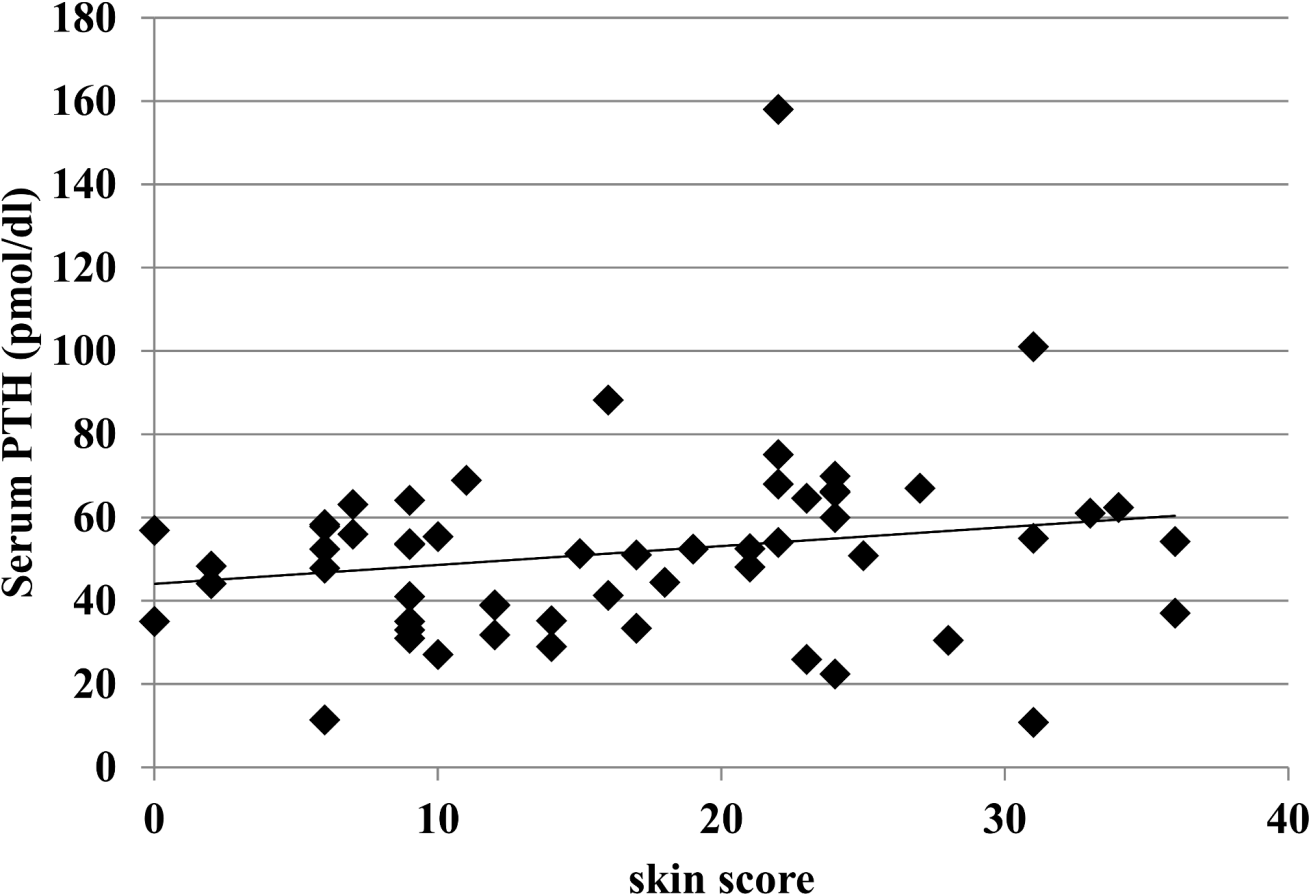
Relationship between the degree of skin fibrosis assessed by modified Rodnan skin score and PTH serum levels in SSc patients. There is no relationship between PTH serum levels and Rodnan skin score, although a tendency to a direct correlation was observed, which did not reach statistical significance.

When measuring the circulating levels of 25OHD in healthy subjects and the two cutaneous subsets of SSc, we found that in all groups they were below the lower limit of normal range levels (30 ng/ml). Nevertheless the circulating levels of 25OHD in healthy subjects (22,93±9,12 ng/ml) were significantly higher compared to the whole group of SSc patients (15,68± 10,18 ng/ml); furthermore, comparing the two cutaneous subset of SSc patients, we found that in dcSSc patients, 25OHD levels were significantly lower compared to the lcSSc ones (11.53±7,46 ng/ml in dcSSc compared to 19,22±11.13 mg/ml in lcSSc, p<0,001—Fig 3). No significant difference in PTH circulating levels was observed between healthy controls, lcSSc and dcSSc. To correlate serum 25OHD and intact PTH levels to skin involvement of SSc patients, we evaluated the extent of skin fibrosis by Rodnan Skin score. We found a significant inverse relationship between the degree of skin fibrosis and circulating levels of 25OHD (r = -0,7 p<0,05, Fig 4); conversely there was no relationship between PTH serum levels and Rodnan skin score, although a tendency to a direct correlation was observed, which did not reach statistical significance (Fig 5).

No difference in serum calcium, phosphorus, alkaline phosphatase, osteocalcin, urine pyridinium cross-links was observed between the three studied groups (S1 Table, S2 Table).

### Relationship between internal organ involvement and BMD, 25OH Vitamin D serum levels and corticosteroid use in limited cutaneous SSc and diffuse cutaneous SSc

No differences were observed concerning internal organ involvement (heart, gastrointestinal, musculo-skeletal, kidney) and disease duration between the limited and the diffuse cutaneous subset of SSc patients excepted for interstitial lung disease whose prevalence was significantly higher in the dSSc subset (p<0,01). A total of 12 (36,4%) lcSSc and 11 dcSSc (31,42%) patients were discontinuously treated with corticosteroids for musculoskeletal symptoms (dosage between 5 and 10 mg/day of prednisone equivalents) for a mean duration of 36 and 42 months years respectively. Only 5 (15,15%) lcSSc and 7 (22,58%) dcSSc received 25–50 mg/day of prednisone equivalents for treatment of lung severe complicances (alveolitis) for a short time (mean duration of treatment 1,2 months, followed by a gradual tapering). No significant differences in the cumulative dose of corticosteroids was detected between lcSSc and and dcSSc.

No significant relationship was found between internal organ involvement (particularly PAH, lung fibrosis, esophageal dysmotility, musculo-skeletal involvement, duration of diseases and malabsorption) and 25OHD serum levels, PTH serum levels, BMI values in the whole group of SSc patients. 25OHD levels were independent from the duration of disease and the presence of anticentromere or antitopoisomerase I autoantibodies.

## Discussion

BMD values and the presence of osteoporosis in SSc patients have been assessed in various reports [15]. Lower BMD values at different sites have been found in patients with SSc compared to healthy controls [2, 12, 16, 17, 18, 19]. Even if attempts have been made to find a correlation between low bone mass and several clinical aspects in SSc patients, the available data regarding the relationship between the condition of ospetopenia/osteoporosis and the extent of skin fibrosis or body mass composition in SSc patients are very discordant [20]. In this report we found that BMD values were significantly lower in SSs post-menopausal patients compared to healthy controls matches for age; further BMD values were significantly lower at both spine and hip in dcSSc compared to lcSSc. No differences in body mass composition (fat and lean mass) between limited and diffuse cutaneous form of diseases and healthy controls were observed, but in contrast with previous published data [21] we found that, in spite of no differences in fat and lean mass, patients with dcSSc showed a significantly lower total mineral content compared to controls and patients with lcSSc.

The reduction of total bone mineral content is associated with a defect in mineralization processes, which in turn could be related to a lower levels of circulating 25OHD due to its well-known role in regulating calcium and phosphorus metabolism, bone formation and mineralization. Further, more recently, in addition to these important metabolic activities, Vitamin D has been recognized to affect the regulation of immune system. Decreased serum levels of 25OHD have been reported in various chronic rheumatic diseases [21–25]. Caramaschi, et al. showed that low levels of vitamin D are very frequent in patients affected by SSc and patients with Vitamin D deficiency presented a more severe disease in comparison with patients with vitamin D insufficiency only [26].

The causes of low serum 25OHD levels in subjects affected by SSc may be various and could include reduced sun exposure due to disability or vascular complications, or intestinal involvement and malabsorption, or renal insufficiency. Skin thickening with capillary damage could also play a role in determining hypovitaminosis D, as it can be involved in a reduced drawing of pre-vitamin D3 synthesized from 7-dehydrocholesterol by UVB radiation in the epidermis. Nevertheless, to date few studies investigate the possible relationship between the extent of skin fibrosis, serum levels of 25OHD and BMD in SSc, with discordant results

In this study, circulating 25OHD was found to be low in control healthy group also, but in SSc patients the observed hypovitaminosis D was more serious; further the levels of 25OHD we found in the diffuse cutaneous form were significantly lower compared to the limited cutaneous form, suggesting that the extent of skin fibrosis could be related to the import of hypovitaminosis D. To correlate serum 25OHD levels to skin involvement, we evaluated skin fibrosis trough Rodnan Skin score and we found a significant inverse relationship between the degree of skin thickness and circulating levels of 25OHD. This data partially agree with a previously published report in which there was no statistical difference in the serum levels of 25OHD between lcSSc and dcSSc, but patients with Rodnan skin score of 10 or less showed a higher mean 25OHD concentration compared with patients with higher score (above 10) [27].

No differences about internal organ involvement were observed between the limited and the diffuse cutaneous subset of SSc (except for interstitial lung disease), even if the prevalence of PAH and lung fibrosis resulted to be higher in respect to what reported in larger cohorts of patients. As the patients included in the presented studied were all post-menopausal women, the high prevalence of PAH could be explained by the older age and a longer disease duration of recruited SSc in respect to the larger systemic sclerosis cohorts which included young patients and patients with early disease. Furthermore it is possible that the duration of disease (defined as years from the first sign/symptoms of disease other than Raynaud phenomenon) is greater than those reported in the **Table 1**, when considering Raynaud phenomenon the first sign of disease. Concerning lung involvement, SSc patients were considered affected by interstitial lung disease also when minimal bi-basal interstitial fibrosis, even if asymptomatic, was detected by lung HRTC. This could explain the high prevalence of interstitial lung disease observed in SSc patients recruited in the study, considering that interstitial abnormalities on HRTC have been reported in up to 90% of SSc patients [28]. We found that the prevalence of interstitial lung disease was significantly greater in dcSSc group, which showed lower BMD and 25OHD serum levels. Data regarding the possible relationship between interstitial lung disease, Vitamin D and bone involvement in SSc are quite discordant. It has been shown that SSc patients with 25OHD levels lower than 10 ng/ml had significantly lower DLCO measurements compared to those with concentrations above 10 ng/ml, but there is conflicting evidence in the previous studies as to whether or not an association between BMD values and lung involvement exist [16, 29].

Also no correlation was found between malabsorption and 25OHD levels/BMD values. Of note that the reported prevalence of malabsorption in patients with SSc can vary widely depending on the case-series and the diagnostic tools employed to evaluate it. The prevalence of SIBO has been reported to be 30–62% in SSc patients and was associated with biochemical signs of subclinical malabsorption [30].

We found no correlation between BMD and 25OHD levels and autoantibody profile, musculo-skeletal involvement, corticosteroid treatment, duration of disease of the whole cohort of SSc patients. These findings are consistent with previous published reports [17,18, 26, 31].

Plasma PTH levels did not show any correlation with the degree of skin fibrosis, even if a non- significant trend toward a direct relationship was observed. Although usually PTH circulating levels inversely correlate with 25OHD levels, the observed data can be explained by the normal levels of serum calcium detected in all SSc recruited patients, as calcium and not 25OHD regulate PTH secretion.

At present, Vitamin D status of SSc patients has not been sufficiently investigated. Even if in some studies a high prevalence of Vitamin D deficiency has been demonstrated among SSc patients, findings concerning the potential correlation between low Vitamin D serum levels and other clinical aspects in SSc patients or the possible determinants of hypovitaminosis D have been inconclusive due to the small size of the studied populations. Further, there is large discrepancy in the literature about BMD and Vitamin D levels in SSc and it is very difficult to compare the different studies on this topic because of the different sample size, the wide age range, the different time course of the recruited patients.

Our results show a significantly lower BMD and total mineral content in the dcSSc subtype; circulating levels of 25OHD were significantly lower in dcSSc patients and inversely correlated with the extent of skin fibrosis. Skin thickness could be one of the factors determining hypovitaminosis D, which in turn could contribute to reduced BMD and total mineral content in these patients.

Further, it should be highlighted that it is well know that Vitamin D affects the immune system at many levels and with different mechanisms, thus deficiency might influence severity and activity disease. Recent studies on murine cells have clearly shown a direct anti-fibrotic effect of Vitamin D. Vitamin D is able to decrease the expression of TGFß1, a pro-fibrotic cytokine, and collagen I and collagen III synthesis in mesenchymal multi-potent cells, and to enhance the expression of some anti-fibrotic factors such matrix metalloproteinase 8 [32]. It has been show that Vitamin D can oppose the effect of TGFß1 on fibroblast proliferation, collagen and fibronectin expression and myofibroblast differentiation [33]. These data lead to hypothesize that there is a link between Vitamin D levels and fibrosis and may furnish an alternative explication for a more severe Vitamin D deficiency in SSc patients with a greater extent of skin fibrosis. To establish if Vitamin D deficiency is a possible causal factor of the worsening fibrotic changes or a consequence of skin fibrosis in SSc it is very difficult and currently there are few published studies on this topic.

The main limitations of the presented report is represented by the relatively small sample size, even it should be highlighted that SSc is a rare disease; further studies are required on the possible causes of Vitamin D deficiency in SSc patients. Studies on the effect of Vitamin D supplementation in SSc patients could confirm the role of Vitamin D not only on BMD and bone metabolism, but also in the in determining the expression various clinical aspect of this disease.

## Supporting Information

S1 Table SSc patients supplementary data. Individual values of SSc groups (dcSSc and lcSSc) of data not shown in the manuscript.
(XLSX)

S2 Table Healthy controls supplementary data. Individual values of healthy controls group of data not shown in the manuscript.
(XLSX)
