# Correction: Relationship between Body Mass Composition, Bone Mineral Density, Skin Fibrosis and 25(OH) Vitamin D Serum Levels in Systemic Sclerosis

**DOI:** 10.1371/journal.pone.0142748

**Published:** 2015-11-06

**Authors:** Addolorata Corrado, Ripalta Colia, Angiola Mele, Valeria Di Bello, Antonello Trotta, Anna Neve, Francesco Paolo Cantatore

Fig 4 appears incorrectly in the published article. Please see the corrected Fig 4 here.

**Fig 4:**
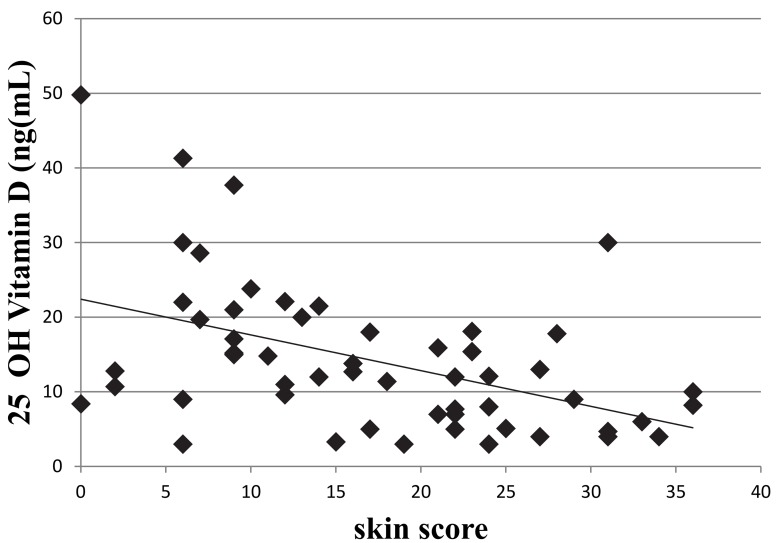
Relationship between the degree of skin fibrosis assessed by modified Rodnan skin score and 25OHD levels in SSc patients. A significant inverse relationship between the degree of skin fibrosis and circulating levels of 25OHD (r = -0.7, p<0.05).
